# cDNA Cloning and Expression Analysis of Pattern Recognition Proteins from the Chinese Oak Silkmoth, *Antheraea pernyi*

**DOI:** 10.3390/insects3041093

**Published:** 2012-10-24

**Authors:** Fengjuan Li, Olle Terenius, Yuan Li, Suyun Fang, Wenli Li

**Affiliations:** 1 School of Life science and Biotechnology, Dalian University of Technology, Dalian, 116023 Liaoning, China; E-Mails: lifengjuan868@163.com (F.L.); ly11_28@yeah.net (Y.L.); fangyun.18@163.com (S.F.); 2 Department of Ecology, Swedish University of Agricultural Sciences (SLU), 750 07 Uppsala, Sweden; E-Mail: olle.terenius@slu.se

**Keywords:** insect immunity, *Antheraea pernyi*, βGRP, lectin, gene expression

## Abstract

Pattern recognition receptors play an important role in insect immune defense. We cloned the β-1,3-glucan recognition protein, lectin-5 and C-type lectin 1 genes of *Antheraea pernyi* and examined the expression profiles of immune-stimulated pupae. After infection with *Bacillus subtilis*, *Escherichia coli*, *Antheraea pernyi* nuclear polyhedrosis virus (*Ap*NPV) and *Saccharomyces cerevisiae*, respectively, the pupae showed different gene expression levels in the different tissues examined (midgut, fatbody, epidermis, testis, and hemocytes). ApβGRP and Aplectin-5 was induced by all the microorganisms, and mainly in epidermis and hemocytes, but not in testis; Aplectin-5 was also expressed in fatbody. Ap C-type lectin 1 was, on the contrary, highly expressed in testis and also in fatbody, but not in hemocytes. Unlike ApβGRP and Aplectin-5, Ap C-type lectin 1 was not induced by Gram-positive bacteria. The results suggest that the cloned lectins may have different functions in different tissues of *A. pernyi*.

## 1. Introduction

Lepidopteran immune resistance to foreign pathogenic microorganisms relies on the recognition of conserved microbial structures of the microorganisms. In turn, this leads to downstream reactions: cellular responses including phagocytosis and nodule formation, humoral responses such as antimicrobial peptide synthesis, and melanization after activation of the prophenoloxidase system [1,2]. For the insect’s humoral immune system, pattern-recognition receptors play an important role. For example, peptidoglycan-recognition proteins can recognize bacterial surfaces and activate relevant immune pathways to resist bacterial invasion. Many other pattern recognition receptors such as hemolin, complement-like proteins and scavenger receptors have also been found in Lepidoptera. 

A β-1,3-glucan recognition protein (βGRP) was originally isolated from the silkmoth *Bombyx mori* by Ochiai *et al.* [3], and has since then been found in other invertebrates, such as the moths *Manduca sexta* [4] and *Plodia interpunctella* [5], and also in crayfish [6] and earthworms [7]. β-GRPs contain a conserved structural domain with a glucan-binding region and can recognize lipopolysaccharide of Gram-negative bacteria, phosphorus acid (of Gram-positive bacteria) and β-1,3-glucan cell walls of fungi, activating immune pathways, and promoting the activation of phenoloxidase [7] and antimicrobial-peptide synthesis [8].

The C-type lectin superfamily is a major type of pattern recognition receptors, which are calcium-dependent carbohydrate-binding proteins [9,10]. They take part in immune responses such as recognition of bacteria and fungi, activation of prophenoloxidase, and hemocyte nodule formation [11,12,13,14,15]. Insect lectins differ from most animal C-type lectins by having two tandem C-type carbohydrate recognition domains (CRDs) instead of one [14,16]. Different CRDs in the same lectin may have different functions [17].

The Chinese oak silkmoth, *Antheraea pernyi*, is an important silk producer, but is commonly struck by infections, which has serious impact on sericulture. A greater understanding of immunity in this species may lead to a selection of more disease-resistant strains. In this paper, the βGRP, lectin-5 and C-type lectin 1 genes of *A. pernyi* were cloned, and their expression profiles following infection with different microorganism were investigated. Also, their *in silico*-deduced secondary structures and microorganism recognition specificities are described. 

## 2. Results and Discussion

### 2.1. cDNA Cloning of ApβGRP, Aplectin-5 and ApCTL1

**Figure 1:**
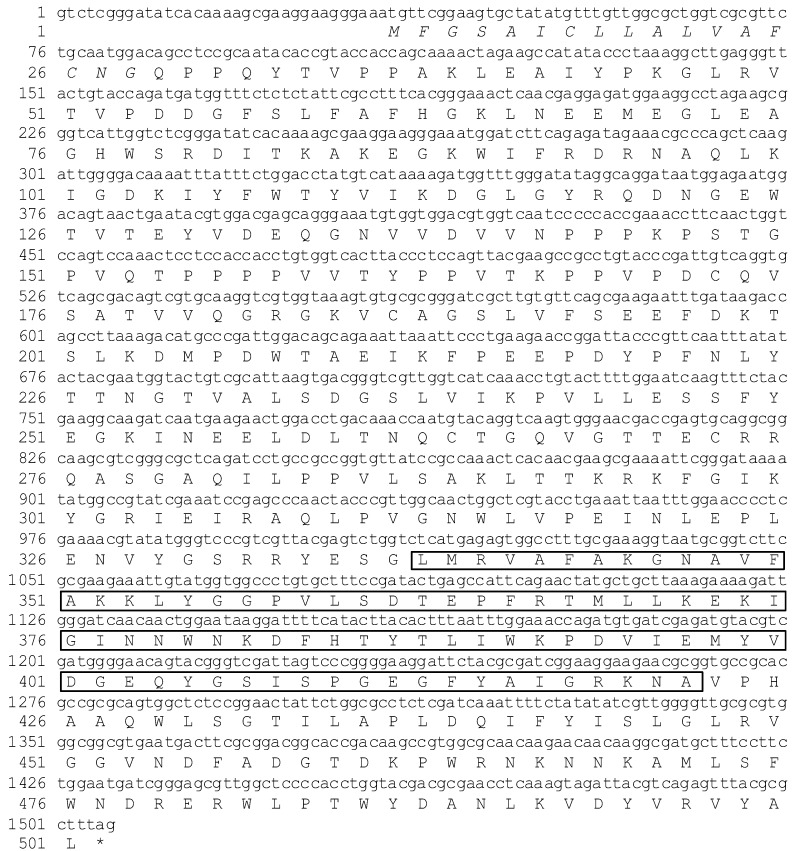
Nucleotide and amino acid sequences of ApβGRP. Italic amino acids indicate the signal peptide of ApβGRP, the boxed amino acids indicate Glyco_hydro_16.

The full length cDNAs of ApβGRP, Aplectin-5 and Ap C-type lectin 1 (ApCTL1) were obtained by RT-PCR after reverse transcription of total RNA of *A. pernyi* fatbody with conserved primers followed by 5’RLM-RACE and 3’RLM-RACE. ApβGRP consists of 2245 nucleotides and has an open reading frame containing 1470 nucleotides (positions 34–1503). Sequence analysis of this protein by a Simple Modular Architecture Research Tool (SMART) suggests that it has a signal peptide of 17 amino acids and contains a conservative glycosidic hydrolase domain belonging to the glycoside hydrolase family 16 (Glyco_hydro_16) (Figure 1), which has the ability to bind β-1,3-glucan.

**Figure 2:**
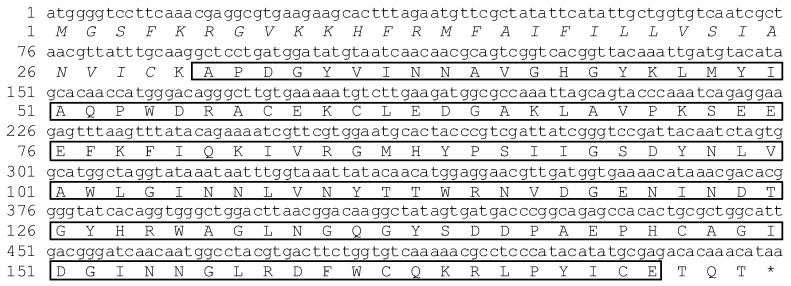
Nucleotide and amino acid sequences of Aplectin-5. Italic amino acids indicate the signal peptide and the boxed amino acids indicate the carbohydrate recognition domain.

The full-length Aplectin-5 cDNA is 589 nucleotides and has an open reading frame (ORF) of 522 bp, which codes for 174 amino acids and with a predicted signal peptide of 29 amino acids (Figure 2).

**Figure 3:**
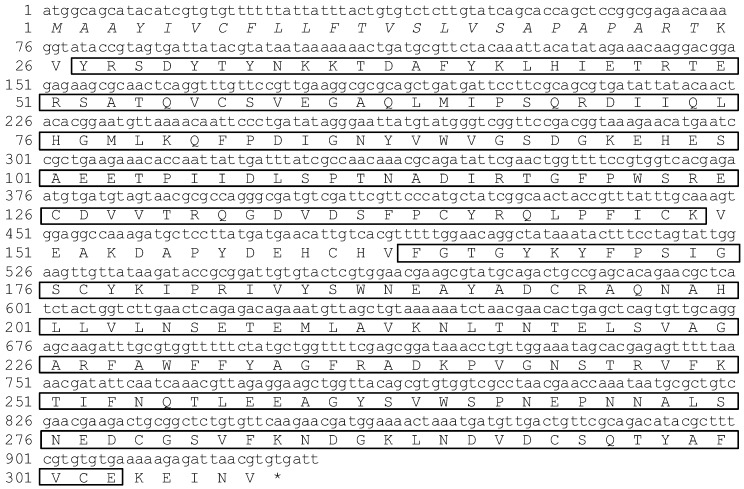
Nucleotide and amino acid sequences of ApCTL1. Italic amino acids indicate the signal peptide and the two sets of boxed amino acids indicate carbohydrate recognition domains.

ApCTL1 has 1035 nucleotides and an open reading frame (ORF) of 924 bp which codes for 308 amino acids with a predicted signal peptide of 24 amino acids (Figure 3). Domain analysis by SMART suggests that these two proteins contain conservative carbohydrate recognition domains (CRDs). However, since ApCTL1 has two CRDs as compared to one in Aplectin-5, we can deduce that Aplectin-5 and ApCTL1 belong to different subfamilies, which also is reflected in Figure 5.

### 2.2. Phylogenetic Analysis of ApβGRP, Aplectin-5 and ApCTL1

**Figure 4:**
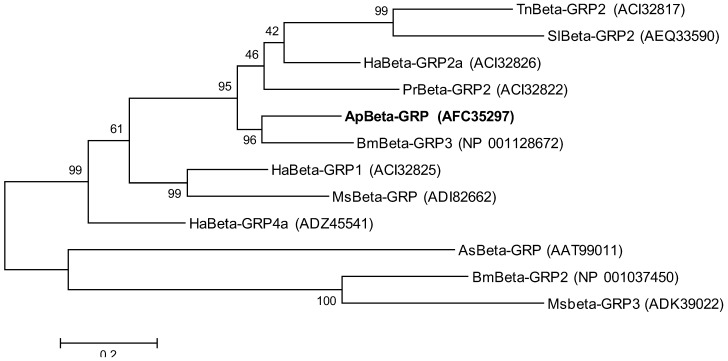
Molecular phylogenetic analysis of β-1,3-glucan recognition proteins (Beta-GRP) from different species. The evolutionary history was inferred by using the Maximum Likelihood method. The tree with the highest log likelihood (–7627.2903) is shown. The percentage of trees in which the associated taxa clustered together is shown next to the branches. The tree is drawn to scale, with branch lengths measured in the number of substitutions per site. Ap, *A. pernyi*; As, *Armigeres subalbatus*; Bm, *B. mori*; Ha, *Helicoverpa armigera*; Hp, *Hepialus pui*; Lo, *Lonomia obliqua*; Ms, *M. sexta*; Pr, *Pieris rapae*; Tn, *Trichoplusia ni*.

To examine the relationship of βGRP from *A. pernyi* with other insect βGRPs, 12 amino acid sequences were used for a phylogenetic tree (Figure 4). In this phylogenetic tree, ApβGRP and *Bombyx mori* βGRP-3 belong to the same branch; the similarity of their amino acid sequences is 83%, which would indicate a similar three-dimensional structure [18]. However, sequence analysis indicates that *Bombyx mori* βGRP-3 has no glucan-binding domain, but instead glycosidase activity, which is another way to recognize and bind to β-1,3-glucan [19]. 

**Figure 5:**
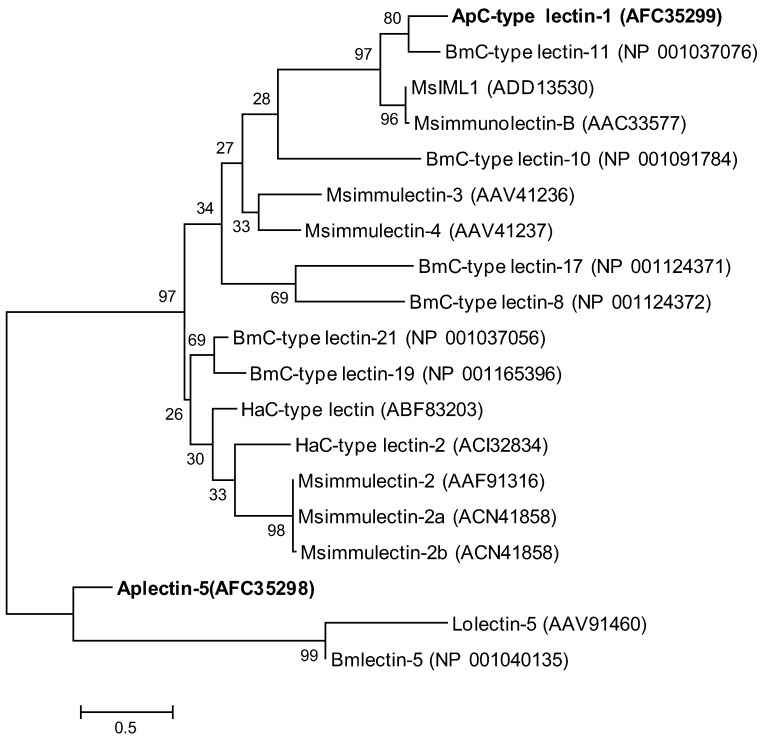
Molecular phylogenetic analysis of lectins from different species. The evolutionary history was inferred by using the Maximum Likelihood method. The percentage of replicate trees in which the associated taxa clustered together in the bootstrap test (1000 replicates) are shown next to the branches [20]. The tree is drawn to scale, with branch lengths measured in the number of substitutions per site. Ap, *A. pernyi*; Bm, *B. mori*; Ha, *Helicoverpa armigera*; Lo, *Lonomia obliqua*; Ms, *M. sexta*.

We also constructed a phylogenetic tree composed of Aplectin-5, ApCTL1 and 17 other lectins from different species (Figure 5). In this phylogenetic tree, *A. pernyi* C-type lectin 1 is most similar to *B. mori* C-type lectin-11, and *M. sexta* IML1 and immunolectin-B. *M. sexta* IML1 is known to bind lipopolysaccharides and activate prophenoloxidase [14] and their similarity of about 60% indicates that they may have a similar function. 

### 2.3. Expression Analysis of ApβGRP, Aplectin-5 and ApCTL1 in Different Tissues

**Figure 6:**
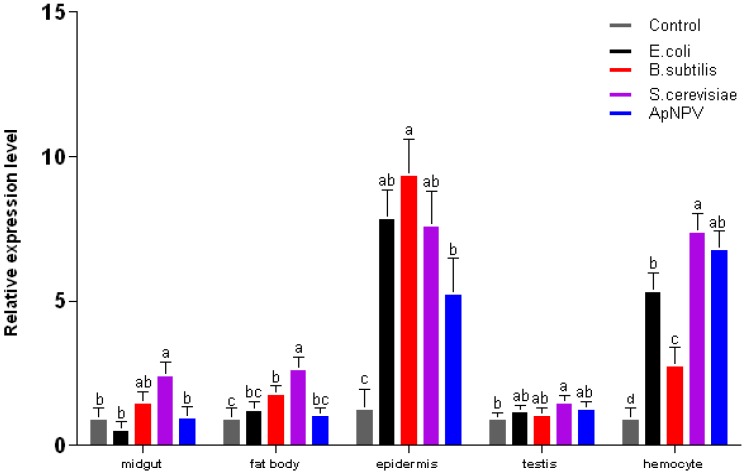
Expression of ApβGRP at 24 h postinjection in different tissues of *A. pernyi* pupae. Different letters indicate significant differences in expression, p < 0.05. Error bars indicate standard deviation of three biological replicates.

In order to identify whether the three pattern recognition genes can be induced by different pathogenic microorganisms, *A. pernyi* diapausing pupae were injected with four different microorganisms: Gram-positive bacteria (*Bacillus subtilis*), Gram-negative bacteria (*Escherichia coli*), *A. pernyi *nuclear polyhedrosis virus (*Ap*NPV), and yeast (*Saccharomyces cerevisiae*). Total RNA was extracted from midgut, fatbody, epidermis, testis and hemocytes 24 h after injection, and quantitative RT-PCR was performed. The expression of ApβGRP was increased by all microorganisms; however, in general, the most significant response was induced by yeast (Figure 6). Also, ApβGRP was expressed mainly in epidermis and hemocytes. 

**Figure 7:**
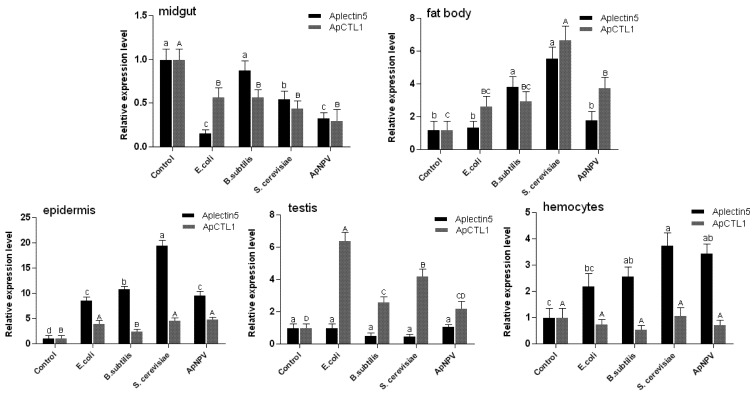
Expression of Aplectin-5 and ApCTL1 at 24 h postinfection in different tissues of *A. pernyi* pupae. Different letters indicate significant differences in expression, p < 0.05. Error bars indicate standard deviation of three biological replicates.

As shown in Figure 7, Aplectin-5 and ApCTL1 can also be induced by all four kinds of microbes, but their expression profiles differ. Aplectin-5, on the one hand, is most up-regulated in epidermis and hemocytes, whereas ApCTL1, on the other hand, has high expression in testis. Both genes were up-regulated in fatbody and down-regulated in the midgut.

These results suggest that ApβGRP, Aplectin-5 and ApCTL1 can participate in relevant immune pathways by recognizing surface components of the four different pathogenic microorganisms. Epidermis is the first line defense of insects, and plays a key role in insect immune systems. All three genes are up-regulated in the epidermis by infection of the four microbes in our experiment, and thus are reacting to an early phase of the infection. In addition, ApβGRP and Aplectin-5 are up-regulated in hemocytes, the second line of defense, which protect the insect through phagocytosis, nodule formation and melanization. ApCTL1 is different from the other two pattern-recognition proteins in that it is expressed in testis and therefore may be important for protecting the next generation

## 3. Experimental Section

### 3.1. Insects, Injection of Microorganisms and Tissue Sample Collections

*Antheraea pernyi* pupae were stored at 4 °C and transferred to room temperature 30 min before injection and then kept at room temperature throughout the experiments. The following microorganisms were used: Gram-positive bacteria (*Bacillus subtilis*), Gram-negative bacteria (*Escherichia coli *Top10), *Antheraea pernyi* nuclear polyhedrosis virus (*Ap*NPV) and fungus (the yeast *Saccharomyces cerevisiae*). 

The pupae were divided into five groups with six pupae in each (three females and three males), four groups were injected 100 μL of one of the following microorganisms: *E. coli *Top10, *B. subtilis*, *S. cerevisiae* (all at OD = 0.8, diluted 1:50 in PBS) or 100 μL *Ap*NPV (2.2 × 10^9^ copies). A control group was injected with PBS only. 24 h after infection, the pupae were sacrificed and dissected into different tissues (epidermis, midgut, fatbody, hemocytes and testis). An equal amount of separated tissue from each pool of three males and three females were immersed together in liquid nitrogen and ground using mortar and pestle. Total RNA was isolated from the ground tissue using TrizolA+ Reagent (Tiangen). 

### 3.2. Cloning of βGRP, Aplectin-5 and ApCTL1 Genes

Total RNA was used to clone the fragments of ApβGRP, Aplectin-5 and ApCTL1 cDNA by RT-PCR (TaKaRa). The primers were designed based on conserved regions of *M. sexta* and *B. mori* as follows:

ApβGRP -F: 5’-CTCAACGAGGARATGGAAGGC-3’,
ApβGRP -R: 5’-ACTGTCCAYTCYCCRTTATCCTG-3’,
ApCTL1-F: 5’- CTACCGTTTATTTGCAAAGTGGAGG -3’,
ApCTL1-R:5’-GTGCTATTTCCAACAGGTTTATCC-3’,
Aplectin-5-F:5’-TCGGGTCCGATTACAATCTAGTG-3’,
Aplectin-5-R: 5’-GTCATCACTATAGCCTTGTCCGT-3’.

Thermal cycle was 94 °C for 5 min, followed by 35 cycles of 94 °C for 30 s, 58 °C for 30 s and 72 °C for 1 min, and a final extension of 72 °C for 7 min. The PCR products were purified by a PCR purification kit (TaKaRa) and cloned into the pMD-18-T vector (TaKaRa). The positive clones were subjected to sequence analysis.

To obtain the full length cDNA of these three genes, 3’RACE and 5’RACE were employed with the FirstChoice RLM-RACE kit (Ambion). The resulting PCR products were purified by a PCR purification kit (TaKaRa), cloned into the pMD-18-T vector and subjected to sequence analysis. The primers for RACE were as follows:

ApβGRP, 5’RACE outer primer: 5’-CTCGGGATATCACAAAAGCGAAGGA-3’
ApβGRP, 5’RACE inner primer: 5’-GCAATACACCGTACCACCAGCAAAA-3’
ApβGRP, 3’RACE outer primer: 5’- GTACCACCAGCAAAACTAGAAGC-3’
ApβGRP, 3’RACE inner primer: 5’- GGTCTCGGGATATCACAAAAGC-3’
Aplectin-5, 5’RACE outer primer: 5’-AACGTTCCTCCATGTTGTATAATTT-3’
Aplectin-5, 5’RACE inner primer: 5’-ACCAAATTATTTATACCTAGCCATG-3’
Aplectin-5, 3’RACE outer primer: 5’-ACAGGTGGGCTGGACTTAACGG-3’
Aplectin-5, 3’RACE inner primer: 5’-AACATGGAGGAACGTTGATGGTGA-3’
ApCTL1, 5’RACE outer primer: 5’-GTTCATCATAAGGAGCATCTTTGGC-3’
ApCTL1, 5’RACE inner primer: 5’-GCCTGTTCCAAAAACGTGACAATGT-3’
ApCTL1, 3’RACE outer primer: 5’-TTTGCGTGGTTTTTCTATGCTGGTT-3’
ApCTL1, 3’RACE inner primer: 5’-ACGCTCATCTACTGGTCTTGAACTC-3’

### 3.3. Phylogenetic Analysis and Protein Structure Prediction

Evolutionary analyses were conducted in MEGA5 [21]. The evolutionary history was inferred by using the Maximum Likelihood method based on the JTT matrix-based model [22]. Initial tree(s) for the heuristic search were obtained automatically as follows. When the number of common sites was <100 or less than one fourth of the total number of sites, the maximum parsimony method was used; otherwise BIONJ method with MCL distance matrix was used. All positions containing gaps and missing data were eliminated. The total number of positions in the final dataset was 369 for Beta-GRPs and 72 for the lectins. SMART online software, http://smart.embl-heidelberg.de [23], was used to predict protein structure.

### 3.4. Transcript Profiles of ApβGRP, Aplectin-5 and ApCTL1

Gene expression levels were measured with real-time quantitative PCR and calculated using the 2^−ΔΔ CT^ method [24] with *A. pernyi* β-actin as internal reference; each type of infection was repeated three times. The primers for transcript profiles were as follows:

Apβ-actin-F: 5’-ACCAACTGGGACGACATGGAGAAA-3’
Apβ-actin-R: 5’-TCTCTCTGTTGGCCTTTGGGTTGA-3’
ApβGRP-F: 5’- ATACGTGGACGAGCAGGGAAATGT-3’
ApβGRP-R: 5’-TTTACCACGACCTTGCACGACTGT-3’
Aplectin-5-F: 5’-CTTCAAACGAGGCGTGAAGAAGCA-3’
Aplectin-5-R: 5’- AATTTGTAACCGTGACCGACTGCG -3’
ApCTL1-F: 5’- AGTGGAGGCCAAAGATGCTCCTTA -3’
ApCTL1-R: 5’- TACGCTTCGTTCCACGAGTACACA -3’

## 4. Conclusions

Pattern recognition receptors play an important role in the insect immune system, they can interact with pathogen-associated molecular patterns (PAMPs) and initiate reactions in the Toll pathways or imd pathways to produce antimicrobial peptides or protease inhibitors for resistance against pathogenic microorganisms [25,26]. In addition, they also regulate cellular immunity to kill microorganisms through phagocytosis, nodule formation and melanization. 

In this study, we cloned the cDNA of ApβGRP, Aplectin-5 and ApCTL1 from the Chinese oak silkmoth, *A. pernyi*. Signal peptide prediction analysis of ApβGRP suggests that the protein is a secretion protein and phylogenetic analysis of ApβGRP and βGRPs from other species showed that the βGRP of *A. pernyi* and the βGRP-3 of *B. mori* belong to the same branch. Though their protein domains are different, both of them can be induced by Gram-negative bacteria, Gram-positive bacteria and fungi, respectively [27]. Their expression is up-regulated in midgut, epidermis and fatbody. The functions of the ApβGRP and the βGRP-3 of *B. mori* may therefore be similar. Protein domain prediction analysis of Aplectin-5 and ApCTL1 showed that they have different structures; Aplectin-5 contains a single CRD while ApCTL1 contains two CRDs, and therefore belong to different subfamilies. Lectins with two or more CRDs can be regarded as an evolutionary innovation by insects, because they can identify more types of microorganisms, and can agglutinate pathogenic microbes thereby improving the efficiency of phagocytosis and nodule formation [28].

ApβGRP and Aplectin-5 were induced by all the microorganisms and mainly in epidermis and hemocytes, but not in testis; Aplectin-5 was also expressed in fatbody. ApCTL1 was on the contrary highly expressed in testis and also in fatbody, but not in hemocytes. Unlike ApβGRP and Aplectin-5 it was not induced by Gram-positive bacteria. The immune system is particularly important in testis because these organs are exposed to the outside environment through the ejaculatory duct and oviduct. In previous investigations, the pattern-recognition protein hemolin was induced in male and female testes of *A. pernyi* pupae after bacterial injection [29] and knock-down of hemolin in Cecropia moths led to lethality in next generation embryos, indicating effects on the reproduction [30]. C-type lectins that are expressed in testes in response to Gram-negative bacteria have previously been found in *B. mori* [13]. Also, in fruit flies, the antimicrobial peptide andropin is present in male genitals [31], and some pattern recognition receptors have been found in generative organ of mammals [32].

In conclusion, we have shown that ApβGRP, Aplectin-5 and ApCTL1 are up-regulated by a variety of pathogens and are differently expressed. The universality emphasizes their important role in the immune system of *A. pernyi*, but their signaling pathways remains to be elucidated.

## References

[1] Jiang H., Vilcinskas A., Kanost M.R. (2010). Immunity in lepidopteran insects. Adv. Exp. Med. Biol..

[2] Yu X.Q., Zhu Y.F., Ma C., Fabrick J.A., Kanost M.R. (2002). Pattern recognition proteins in *Manduca sexta* plasma. Insect Biochem. Mol. Biol..

[3] Ochiai M., Ashida M. (1988). Purification of a beta-1,3-glucan recognition protein in the prophenoloxidase activating system from hemolymph of the silkworm, *Bombyx mori*. J. Biol. Chem..

[4] Jiang H.B., Ma C.C., Lu Z.Q., Kanost M.R. (2004). B-1,3-glucan recognition protein-2 (beta grp-2) from *Manduca sexta*: An acute-phase protein that binds beta-1,3-glucan and lipoteichoic acid to aggregate fungi and bacteria and stimulate prophenoloxidase activation. Insect Biochem. Mol. Biol..

[5] Fabrick J.A., Baker J.E., Kanost M.R. (2003). cDNA cloning, purification, properties, and function of a beta-1,3-glucan recognition protein from a pyralid moth, *Plodia interpunctella*. Insect Biochem. Mol. Biol..

[6] Duvic B., Söderhäll K. (1990). Purification and characterization of a beta-1,3-glucan binding-protein from plasma of the crayfish *Pacifastacus leniusculus*. J. Biol. Chem..

[7] Beschin A., Bilej M., Hanssens F., Raymakers J., Van Dyck E., Revets H., Brys L., Gomez J., De Baetselier P., Timmermans M. (1998). Identification and cloning of a glucan- and liopoplysaccharide-binding protein from *Eisenia foetida* earthworm involved in the activation of prophenoloxidase cascade. J. Biol. Chem..

[8] Kim Y.S., Ryu J.H., Han S.J., Choi K.H., Nam K.B., Jang I.H., Lemaitre B., Brey P.T., Lee W.J. (2000). Gram-negative bacteria-binding protein, a pattern recognition receptor for lipopolysaccharide and beta-1,3-glucan that mediates the signaling for the induction of innate immune genes in *Drosophila melanogaster* cells. J. Biol. Chem..

[9] Vasta G.R., Quesenberry M., Ahmed H., O'Leary N. (1999). C-type lectins and galectins mediate innate and adaptive immune functions: Their roles in the complement activation pathway. Dev. Comp. Immunol..

[10] Weis W.I., Taylor M.E., Drickamer K. (1998). The C-type lectin superfamily in the immune system. Immunol. Rev..

[11] Ling E.J., Ao J.Q., Yu X.Q. (2008). Nuclear translocation of immulectin-3 stimulates hemocyte proliferation. Mol. Immunol..

[12] Ling E.J., Yu X.Q. (2006). Cellular encapsulation and melanization are enhanced by immulectins, pattern recognition receptors from the tobacco hornworm manduca sexta. Dev. Comp. Immunol..

[13] Takase H., Watanabe A., Yoshizawa Y., Kitami M., Sato R. (2009). Identification and comparative analysis of three novel C-type lectins from the silkworm with functional implications in pathogen recognition. Dev. Comp. Immunol..

[14] Yu X.Q., Gan H., Kanost M.R. (1999). Immulectin, an inducible C-type lectin from an insect, *Manduca sexta*, stimulates activation of plasma prophenol oxidae. Insect Biochem. Mol. Biol..

[15] Yu X.Q., Kanost M.R. (2000). Immulectin-2, a lipopolysaccharide specific lectin from an insect, *Manduca sexta*, is induced in response to Gram-negative bacteria. J. Biol. Chem..

[16] Koizumi N., Imamura M., Kadotani T., Yaoi K., Iwahana H., Sato R. (1999). The lipopolysaccharide-binding protein participating in hemocyte nodule formation in the silkworm *Bombyx mori* is a novel member of the C-type lectin superfamily with two different tandem carbohydrate-recognition domains. FEBS. Lett..

[17] Shin S.W., Park D.S., Kim S.C., Park H.Y. (2000). Two carbohydrate recognition domains of *Hyphantria cunea* lectin bind to bacterial lipopolysaccharides through o-specific chain. FEBS. Lett..

[18] Chothia C., Lesk A.M. (1986). The relation between the divergence of sequence and structure in proteins. EMBO. J..

[19] Takahasi K., Ochiai M., Horiuchi M., Kumeta H., Ogura K., Ashida M., Inagaki F. (2009). Solution structure of the silkworm betagrp/gnbp3 n-terminal domain reveals the mechanism for beta-1,3-glucan-specific recognition. P. Natl. Acad. Sci. USA.

[20] Felsenstein J. (1985). Confidence-limits on phylogenies - an approach using the bootstrap. Evolution.

[21] Tamura K., Peterson D., Peterson N., Stecher G., Nei M., Kumar S. (2011). MEGA5: Molecular evolutionary genetics analysis using maximum likelihood, evolutionary distance, and maximum parsimony methods. Mol. Biol. Evol..

[22] Jones D.T., Taylor W.R., Thornton J.M. (1992). The rapid generation of mutation data matrices from protein sequences. Comput. Appl. Biosci..

[23] Letunic I., Doerks T., Bork P. (2012). SMART 7: Recent updates to the protein domain annotation resource. Nucleic Acids Res..

[24] Livak K.J., Schmittgen T.D. (2001). Analysis of relative gene expression data using real-time quantitative pcr and the 2(t)(-delta delta c) method. Methods.

[25] Hoffmann J.A. (2003). The immune response of drosophila. Nature.

[26] Medzhitov R., Janeway C.A. (2002). Decoding the patterns of self and nonself by the innate immune system. Science.

[27] Xu P.-Z., Zhang M.-R. (2010). Molecular cloning and expression profile analysis of genes encoding pattern recognition receptors pgrp and βgrp in the silk-worm *Bombyx mori*. Sci. Sericulture.

[28] Yu X.Q., Kanost M.R. (2003). *Manduca sexta* lipopolysaccharide-specific immulectin-2 protects larvae from bacterial infection. Dev. Comp. Immunol..

[29] Li W., Terenius O., Hirai M., Nilsson A.S., Faye I. (2005). Cloning, expression and phylogenetic analysis of hemolin, from the chinese oak silkmoth, *Antheraea pernyi*. Dev. Comp. Immunol..

[30] Bettencourt R., Terenius O., Faye I. (2002). Hemolin gene silencing by ds-RNA injected into cecropia pupae is lethal to next generation embryos. Insect Mol. Biol..

[31] Samakovlis C., Kylsten P., Kimbrell D.A., Engstrom A., Hultmark D. (1991). The andropin gene and its product, a male-specific antibacterial peptide in *Drosophila melanogaster*. EMBO. J..

[32] Phatsara C., Jennen D.G.J., Ponsuksili S., Murani E., Tesfaye D., Schellander K., Wimmers K. (2007). Molecular genetic analysis of porcine mannose-binding lectin genes, mbl1 and mbl2, and their association with complement activity. Int. J. Immunogenet..

